# Cervical Cystic Hygroma in an Adult

**DOI:** 10.1155/2014/209427

**Published:** 2014-12-07

**Authors:** Serhan Derin, Murat Şahan, Yelda Dere, Neşat Çullu, Leyla Şahan

**Affiliations:** ^1^Department of Otolaryngology, Muğla Sıtkı Koçman University School of Medicine, Orhaniye Mahallesi, Haluk Özsoy Caddesi, 48000 Mugla, Turkey; ^2^Department of Pathology, Muğla Sıtkı Koçman University School of Medicine, 48000 Mugla, Turkey; ^3^Department of Radiology, Muğla Sıtkı Koçman University School of Medicine, 48000 Mugla, Turkey; ^4^Department of Anesthesiology and Reanimation, Muğla Sıtkı Koçman University School of Medicine, 48000 Mugla, Turkey

## Abstract

Cystic hygromas/lymphangiomas are extremely rare malformations in adults. They are usually seen in infants and children under 2 years of age. En bloc resection is difficult due to the adhesive characteristics of the tumors. Inadequate surgical intervention often leads to recurrent disease. We report herein the case of a cystic hygroma/lymphangioma that presented as an uncommon mass on the cervical region in an adult, together with its histopathological, radiologic, and operative features.

## 1. Introduction

Cystic hygromas/lymphangiomas are rare congenital malformations of the lymphatic system. Most are seen in the head and neck region (75–80%) and usually affect children under 2 years of age [1]. They are quite rare in adults [2]. The etiology of hygromas in adults is controversial, but they are thought to be due to proliferation of lymphoid vessels in response to head and neck trauma and/or infection [3]. Here, we present the case of a 38-year-old male patient with cystic hygroma in the cervical region.

## 2. Case Report

**Figure 1:**
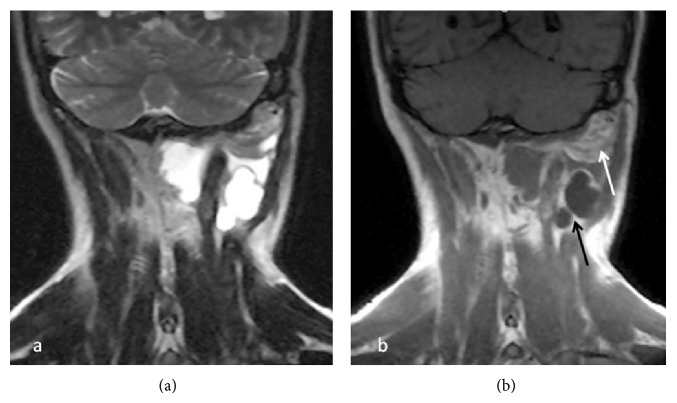
MRI findings of a 38-year-old male showed a multilobular cystic mass that was 8 × 5 × 4 cm and extended from the mastoid bone's posterior border along the sternocleidomastoid muscle to the larynx and was hyperintense on T2-weighted images (a) and hypointense on postcontrast T1-weighted images (b).

**Figure 2:**
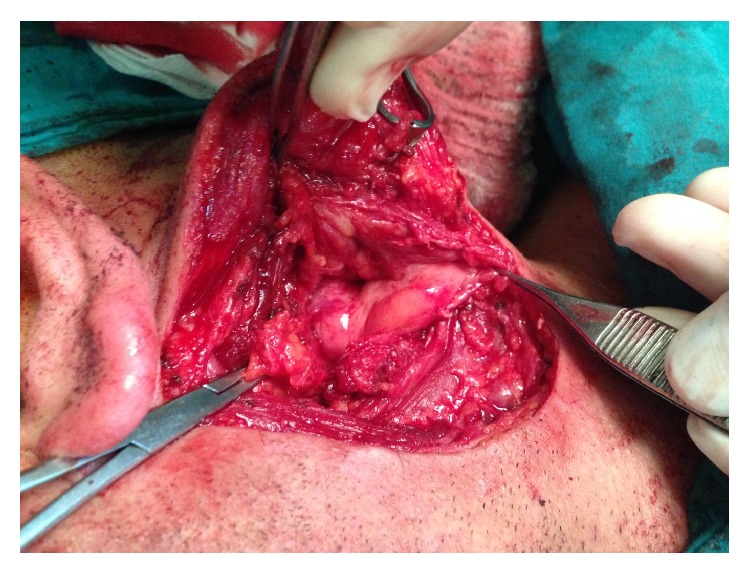
The cystic mass was located between the posterior border of the left sternocleidomastoid muscle and the mastoid apex.

**Figure 3:**
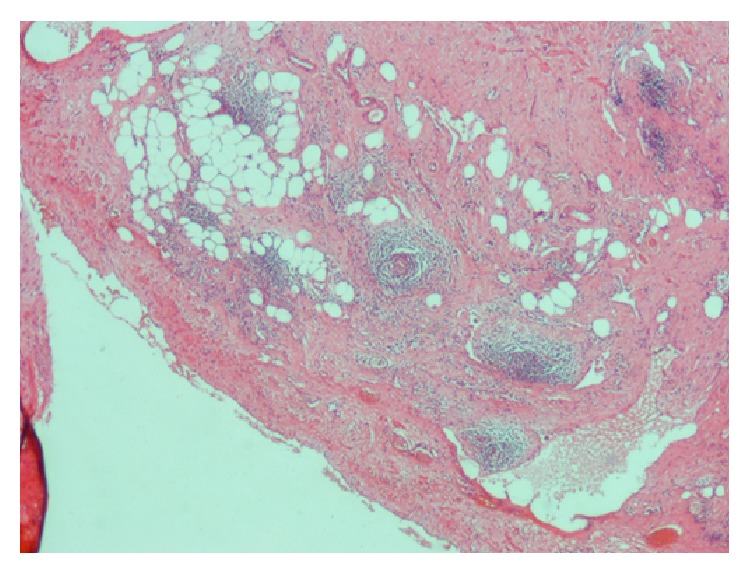
The tumor had a large vascular space lined by flattened epithelium and lymphoid aggregates in the surrounding fibroblastic stroma (H&E, ×200).

**Figure 4:**
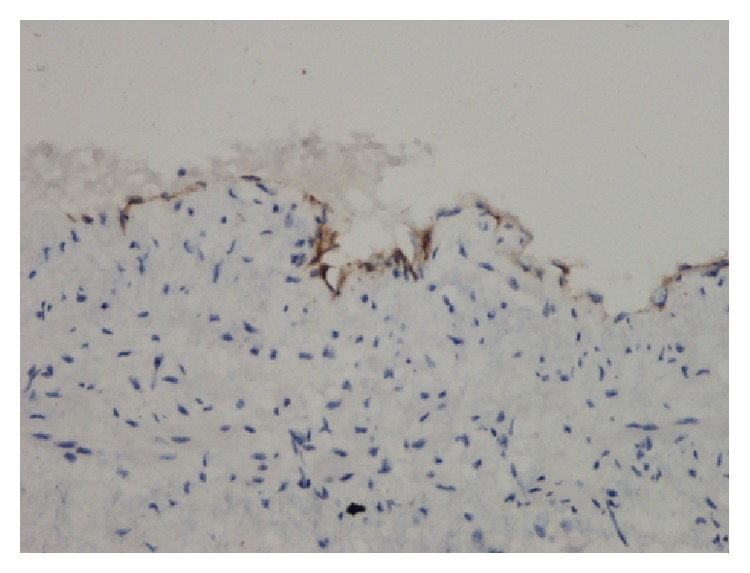
D2-40 immunostaining, DAB, ×200.

**Figure 5:**
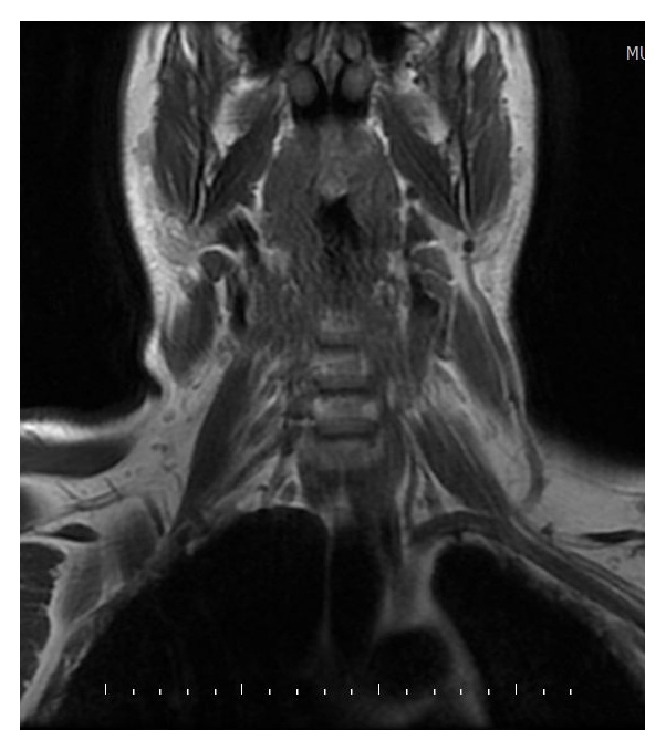
MRI was normal at the postoperative period.

A 38-year-old male presented with a painful left suboccipital cystic mass and a history of recurrent infection episodes for 6 months. During examination, a soft, smooth, and compressible 3 × 3 cm cystic mass was detected in the suboccipital region. Ultrasonographic findings revealed a multilobular cystic mass that extended from the suboccipital region to the postauricular region. Magnetic resonance imaging (MRI) findings showed a multilobular cystic mass with a size of 8 × 5 × 4 cm extending from the posterior border of the mastoid bone along the sternocleidomastoid muscle to the larynx and hyperintense on T2-weighted images (Figure 1(a)) and hypointense on postcontrast T1-weighted images (Figure 1(b)). Fine-needle aspiration cytologic diagnosis demonstrated a cystic lesion, and the diagnosis was lymphangioma. During surgery, extreme adherence of the cyst wall to adjacent structures, especially the mastoid bone, sternocleidomastoid muscle, spinal accessory nerve, and posterior cervical spinal nerves, was observed (Figure 2). The cyst wall extended to the posterior margin of the cervical vertebra. Due to its wide extension and vital tissue contiguity, the dissection was difficult and the cyst was excised totally piecemeal without any vital structure damage. Histopathologic findings revealed a large vascular space containing blood and lined by flattened endothelium. The lining endothelium consisted of small endothelial cells commonly flattened. Around these vascular structures, small lymphoid aggregates were detected in the surrounding fibroblastic stroma (Figure 3). Immunohistochemically, CD31, CD34, and D2-40 were positive and pancytokeratin was negative in the lining endothelium of the cysts (Figure 4). These findings confirmed the diagnosis of cystic hygroma. In postoperative follow-up, the patient was asymptomatic during the following 11-month period and MRI was normal (Figure 5).

## 3. Discussion

Cystic hygromas/lymphangiomas are thought to be developmental abnormalities associated with a failure in the embryological connection between lymphoid vessels and venous system and generally not accepted as true tumors [4]. Three types of cystic hygroma/lymphangioma can be distinguished. The capillary form is usually asymptomatic with small sizes. Although cavernous and cystic lymphangiomas show the same histological pattern, cystic lesions are usually larger and symptomatic [5].

Microscopically, lymphangioma is characterized by large, dilated lymphatic vessels in a fibrotic or loose stromal background. Focal areas of papillary endothelial proliferation were described aside by the classic histological picture [6]. The presence of lymphoid aggregates even lymphoid follicles may be confusing when existing in large amounts and needs to be distinguished from atypical lymphoid proliferations. The main histopathologic differential diagnosis of cystic hygroma is cavernous hemangioma in which blood filled large cystic spaces were described to be similar to lymphangioma. The lining endothelium of cavernous hemangioma shows positivity with pancytokeratin and Factor VIII, immunohistochemically. The presence of lymphatic spaces with thin walls containing fibrous tissue, smooth muscle, and lymphoid aggregates favors the diagnosis of lymphangioma [7]. In general, lymphangioma is accepted as a benign tumor with no malignant transformation and curable by excision [8].

Primary treatment for lymphangioma is total surgical excision [9], but, due to the invasive characteristics of cystic hygromas, en bloc resection is difficult especially in the head and neck region. Imaging studies are important to assess extension of the lesion. Although ultrasound scanning is sufficient to establish the diagnosis, computed tomography (CT) or MRI is useful to show adjacent tissue extension [5]. In our case, the cyst was fairly adherent to adjacent tissue, which included the sternocleidomastoid muscle, spinal accessory nerve, and posterior cervical spinal nerves. Therefore, we preferred piecemeal excision to avoid adjacent tissue damage. Total excision is important for the prognosis, because cyst remnants usually cause tumor recurrence. In addition, it should be kept in mind that there are important complications of surgical therapy of cystic hygromas in the head and neck region such as cranial nerve injury [10].

On the other hand, there are several nonsurgical treatment modalities for cystic hygroma such as interferon alpha, laser therapy, and intralesional sclerosing agents administration [11, 12].

OK 432 is one of the most commonly used sclerosing agents. It is produced by group A* Streptococcus pyogenes*. It produces an inflammatory reaction when applied with intracystic injection. Then it causes the destruction of endothelium, sclerosis, and cicatricial contraction of cyst wall. Although success rate is variable, there are promising results. For instance, complete or marked regression is reported in 10 of 11 patients with OK 432 administration for cystic hygroma in the study of Laranne et al. Depending on these results, they think that OK 432 should be used as the primary form of treatment for lymphangiomas especially in children due to good results and minimal complication rate [10]. Fever and local inflammatory reaction are the common side effects of OK 432 [11, 13, 14]. But hypopharyngeal edema was reported especially on administration for cysts located near the airway [10].

Laser therapy reduces cyst size but it has significant risk of damage to the overlying skin [15]. Finally, interferon alpha is used on lymphangioma and hemangioma by means of antiangiogenic effect. The most common side effects of interferon alpha are fever, neutropenia, and diarrhea [12].

As a result of the aforementioned characteristics, cystic hygromas are rather rare malformations in adults. However, it should be considered in the differential diagnosis of head and neck cystic masses in adults. Surgeons should ensure that total resection of the tumor without any remnant is performed and that the unity of vital tissues is conserved during surgery.
